# Dual RNAseq analyses at soma and germline levels reveal evolutionary innovations in the elephantiasis-agent *Brugia malayi*, and adaptation of its *Wolbachia* endosymbionts

**DOI:** 10.1371/journal.pntd.0008935

**Published:** 2021-01-06

**Authors:** Germain Chevignon, Vincent Foray, Mercedes Maria Pérez-Jiménez, Silvia Libro, Matthew Chung, Jeremy M. Foster, Frédéric Landmann

**Affiliations:** 1 CRBM, University of Montpellier, CNRS, Montpellier, France; 2 Laboratoire de Génétique et Pathologie des Mollusques Marins, Ifremer, La Tremblade, France; 3 Institut de Recherche sur la Biologie de l’Insecte, UMR 7261, CNRS, Université de Tours, Tours, France; 4 Centro Andaluz de Biología del Desarrollo (CABD)–Universidad Pablo de Olavide (UPO), Departamento de Biología Molecular e Ingeniería Bioquímica, UPO/CSIC/JA, Sevilla, Spain; 5 Division of Protein Expression & Modification, New England Biolabs, Ipswich, Massachusetts, United States of America; 6 Institute for Genome Sciences, University of Maryland School of Medicine, Baltimore, Maryland, United States of America; 7 Department of Microbiology and Immunology, University of Maryland School of Medicine, Baltimore, Maryland, United States of America; University of Glasgow, UNITED KINGDOM

## Abstract

*Brugia malayi* is a human filarial nematode responsible for elephantiasis, a debilitating condition that is part of a broader spectrum of diseases called filariasis, including lymphatic filariasis and river blindness. Almost all filarial nematode species infecting humans live in mutualism with *Wolbachia* endosymbionts, present in somatic hypodermal tissues but also in the female germline which ensures their vertical transmission to the nematode progeny. These α-proteobacteria potentially provision their host with essential metabolites and protect the parasite against the vertebrate immune response. In the absence of *Wolbachia wBm*, *B*. *malayi* females become sterile, and the filarial nematode lifespan is greatly reduced. In order to better comprehend this symbiosis, we investigated the adaptation of *wBm* to the host nematode soma and germline, and we characterized these cellular environments to highlight their specificities. Dual RNAseq experiments were performed at the tissue-specific and ovarian developmental stage levels, reaching the resolution of the germline mitotic proliferation and meiotic differentiation stages. We found that most *wBm* genes, including putative effectors, are not differentially regulated between infected tissues. However, two *wBm* genes involved in stress responses are upregulated in the hypodermal chords compared to the germline, indicating that this somatic tissue represents a harsh environment to which *wBm* have adapted. A comparison of the *B*. *malayi* and *C*. *elegans* germline transcriptomes reveals a poor conservation of genes involved in the production of oocytes, with the filarial germline proliferative zone relying on a majority of genes absent from *C*. *elegans*. The first orthology map of the *B*. *malayi* genome presented here, together with tissue-specific expression enrichment analyses, indicate that the early steps of oogenesis are a developmental process involving genes specific to filarial nematodes, that likely result from evolutionary innovations supporting the filarial parasitic lifestyle.

## Author summary

Parasitic filarial nematodes affect over 100 million individuals in tropical areas where they cause debilitating diseases. Almost all species infecting humans live in mutualism with *Wolbachia* endosymbionts. The *Wolbachia wBm* of *Brugia malayi*, a nematode responsible for human elephantiasis, show a typical localisation in the hypodermis of the adult worms, and in the female germline in order to be vertically transmitted to the next generation through the eggs. Analyses of *Wolbachia*-depleted filarial nematodes have revealed that these α-proteobacteria contribute to the long lifespan of the nematode host. In addition, they clearly support the maintenance of the female germline, indicating a developmental symbiosis between *B*. *malayi* and its associated *wBm* endosymbiont. To understand the mutualism between *wBm* and the *B*. *malayi* host, we performed transcriptomics analyses at an unprecedented tissue-specific resolution, during oogenesis and in the soma, and we characterized the adaptation of *wBm* to specific cellular environments such as the hypodermis. Additional comparative germline transcriptomic analyses between *B*. *malayi* and *C*. *elegans* as well as a *B*. *malayi* orthology map revealed peculiarities in the filarial oogenesis, likely resulting from evolutionary innovations specific to the parasitic lifestyle.

## Introduction

With an estimated excess of a million species, the phylum Nematoda alone constitutes ~80% of the multicellular organisms on earth, with its members having colonized all regions of the world [1]. The phylum is divided into five clades, presenting a broad variety of phenotypic traits with differing lifespans, body lengths, modes of reproduction and habitats, accompanied by significant genomic diversity, reflected in a genome size ranging from 42 to 700 Mb [2]. In addition, the evolutionary history of nematodes that typically feed on microorganisms has favored symbiotic associations with alpha or gamma-proteobacteria [3]. While most species live in soil or water, some have become parasites of animals or plants. Parasitism has emerged in Nematoda >15 times, across four different clades [4]. Notably, clade III harbors parasitic species engaged in mutualistic symbiosis. It encompasses the family Onchocercidae, also called filariae or filarial nematodes. Their third larval stage is infective and transmitted by haematophagous arthropods to vertebrate hosts, in which adult filarial worms reproduce predominantly in the lymphatics, subcutaneously, or in the serous cavity [5]. Females produce numerous progeny, the microfilariae, which continue the life cycle in the arthropod vector only, after a blood-feed. About 40% of all filarial species live in association with *Wolbachia* endosymbionts [6], a genus of intracellular obligate alpha-proteobacteria widely present among arthropods, but limited to the Onchocercidae family in animal parasitic nematodes [7]. The *Wolbachia* represent a monophyletic bacterial genus sub-divided into supergroups, with some specific to filarial nematodes [7,8]. Within a supergroup, each *Wolbachia* strain has its genomic specificities, and phylogenetic studies have revealed a strong co-evolution between *Wolbachia* and their filarial host species [6].

Current views on the history of this symbiosis suggest independent *Wolbachia* acquisitions in filarial ancestors, as well as secondary losses [8]. The *Wolbachia* are harbored by seven out of eight filarial species causing human filariasis, a group of debilitating and chronic neglected diseases in tropical areas, and other diseases that affect cattle, canids, and felids [9]. Regardless of their invertebrate host species, the *Wolbachia* are transmitted vertically through the female germline to the next generation. In addition to the female gonads, these endosymbionts also populate the somatic hypodermal chords in both male and female worms, a tissue that secretes the worm's cuticle and filters nutrients from the vertebrate host [10,11]. When filarial nematodes are depleted of their *Wolbachia* by antibiotic therapy, larval molting is interrupted and adult females become sterile. This loss of *Wolbachia* is also macrofilaricidal, unlike any classical anthelminthic drug currently in use, since the adult nematode lifespan is dramatically reduced from >10 years to months [12]. In the context of emerging anthelminthic drug resistance, biomedical research is focusing on the search for both new anti-filarials and potent anti-*Wolbachia* compounds [9,13–19].

Because of their strong host specificity, it is impossible to maintain human-specific filarial species in the laboratory with the exception of *Brugia malayi*, an agent of human elephantiasis whose complete life cycle can be maintained in laboratory conditions with *A*. *aegypti* mosquito vectors and the Mongolian gerbil as a surrogate host. Hence *B*. *malayi* and its associated *wBm Wolbachia* genomes were the first ones to be sequenced among filarial species [20,21].

Several hypotheses have been formulated to explain the mutualism between *Wolbachia* and filarial nematodes. First, the presence of *Wolbachia* modulates the Th2-type vertebrate immune response towards filarial nematodes, which would otherwise lead to a fatal eosinophil degranulation of the worm's cuticle, suggesting a defensive mutualism [22]. Second, genomic and transcriptomic studies performed on several filarial species highlight incomplete biosynthesis pathways in both the nematode host and the bacterial endosymbionts, and support a possible mutual nutritional provisioning [21,23,24]. *B*. *malayi* lacks the capacity to produce haem, FAD, and riboflavin cofactors and lacks the capacity for *de novo* synthesis of nucleotides, while *wBm* is autotrophic for nucleotide synthesis but unable to synthesize most amino acids. However, how the *Wolbachia* support filarial longevity and homeostasis remains unclear. Without ruling out metabolic mutualism, the genome of the *Wolbachia*-free filarial nematode *Loa loa* reveals the same metabolic limitations compared to *B*. *malayi*, suggesting additional or more subtle contributions or subversions from *Wolbachia* [25].

These endosymbionts are also involved in a developmental symbiosis regarding the germline. During *in utero* embryonic development, *Wolbachia* asymmetrically segregate to specific blastomeres in order to colonize precursors of hypodermal tissues. In juvenile females, the endosymbionts show an ovarian tropism from the hypodermal chords to the developing distal germline, which they populate at high titers [10,26]. Upon *Wolbachia* depletion by antibiotic treatment, a massive apoptosis occurs during embryogenesis [27], preceded by polarity defects in early embryos, suggesting the production of oocytes defective for maternal products [28], indicating the absence of endosymbionts dramatically impacts the germline.

The ovary of *B*. *malayi* is organized similarly to the gonad of the free-living *C*. *elegans* nematode, whose germline development has been extensively studied. A pool of germline stem cell nuclei organized as a syncytium gives rise to progenitors that proliferate mitotically in the distal part of the tube-shaped ovary, capped by a distal tip cell (DTC). In both species, proliferation is promoted by a Notch signaling pathway, that has been shown in *C*. *elegans* to prevent the meiotic switch, through post-transcriptional inhibition of meiotic genes [29]. Next, these nuclei are displaced away from the Notch distal source and differentiate to commit to the meiotic program. In *B*. *malayi*, oocytes grow and cellularize during meiotic prophase I, before entering the uterus where they are fertilized. Without *wBm*, the *B*. *malayi* female germline mitotic proliferation is reduced by a third, and activity and organization of the germline stem cells (GSCs) are perturbed [30]. This demonstrates that *wBm* are necessary for maintenance of a functional germline that relies heavily on its endosymbionts in the earliest steps of oogenesis, in the proliferative zone. *Wolbachia* possess a functional type IV secretion system, that allows them to secrete potential effectors able to subvert the host biology to promote their survival and replication [24,31]. It is not clear yet whether *wBm* can differentially express genes, including putative effectors, between somatic and germline tissues, and during the different stages of oogenesis. It is also important to understand their cellular environments, not only to identify potential drug targets, but to better understand this unique developmental symbiosis. Although the experimental paradigm of *C*. *elegans* teaches us a lot about stem cell and germline biology, this short-lived nematode offers limited insight on the making of the germline in *B*. *malayi*, a parasitic species estimated phylogenetically distant by ~350 My [32], and producing millions of embryos during an impressive lifespan. We report transcriptomic studies using dual RNAseq approaches at tissue-specific, and germline developmental stage-specific levels, to define the transcriptomes of *wBm* and its nematode host, *B*. *malayi*. In addition, comparative germline transcriptomics with *C*. *elegans* together with the first *B*. *malayi* genome orthology maps indicate that germline stem cells and their proliferation extensively rely on evolutionary innovations specific to filarial nematodes, while the meiotic program shares more conservation with *C*. *elegans*.

Among the vast majority of *Wolbachia* genes not differentially regulated between germline and somatic sources, the most expressed genes are related to protein synthesis, ATP synthesis and pyruvate catabolism, suggesting an apparent similar *wBm* growth rate between tissues. Few highly upregulated *wBm* genes related to stress specifically in the hypodermis suggest that this tissue may constitute a challenging environment.

## Results

### Dual-RNAseq approaches at soma and germline levels

**Fig 1:**
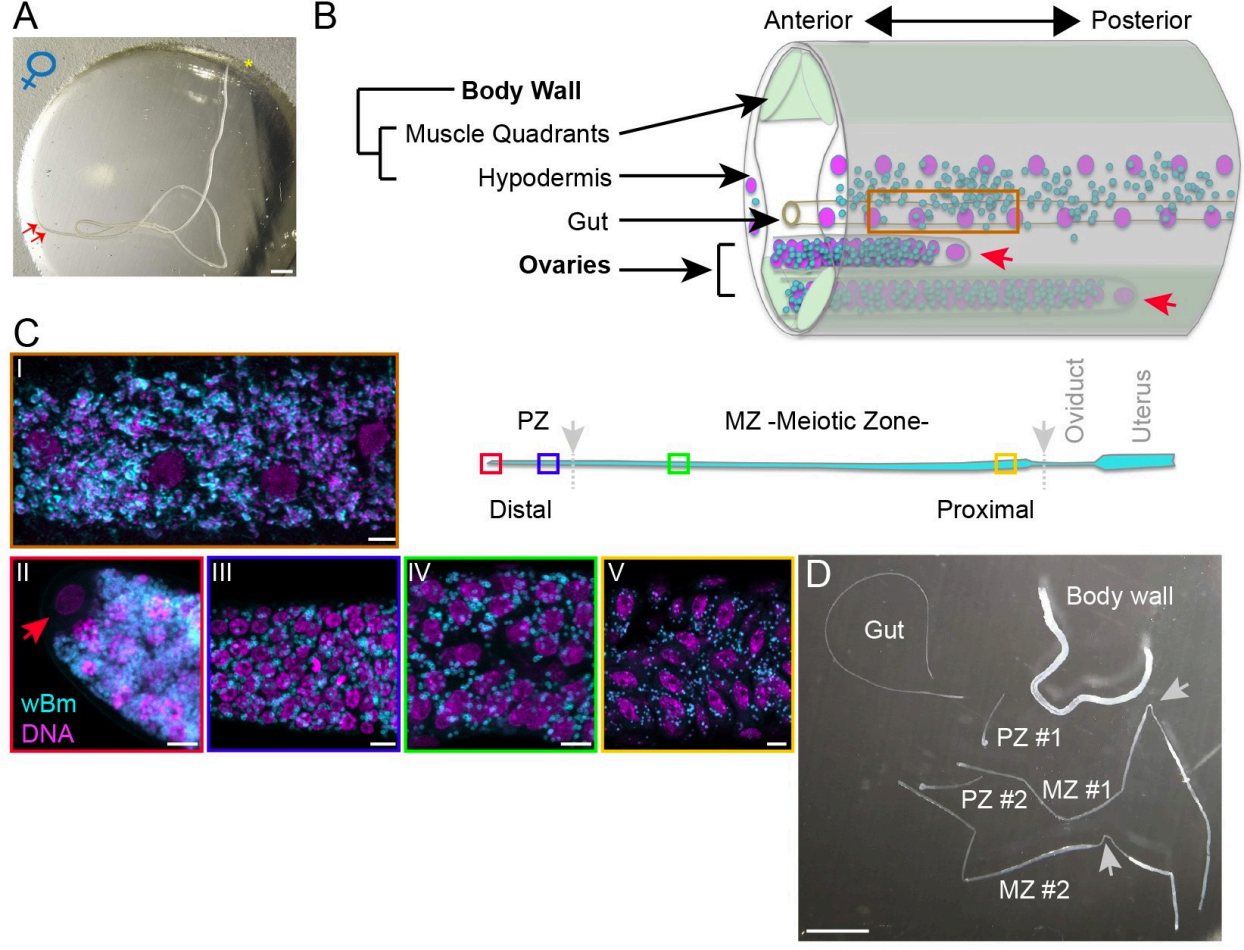
Isolation of the germline and somatic tissues from a *Brugia malayi* female. (A) A female *Brugia malayi* prior to dissection in a glass spot plate. The asterisk indicates the anterior part. The two arrows point towards the distal parts of the ovaries. Scale bar = 1 mm. (B) Schematic drawing of a female cross-section in the posterior. Nuclei are represented in magenta and *wBm* in cyan. The two red arrowheads indicate the distal part of the ovaries capped by a distal tip cell -DTC-. (C) Schematic drawing of an ovary, and confocal images corresponding to the tissues or germline developmental stages sampled for the RNAseq analyses. DNA is revealed by DAPI in magenta and an anti-WSP antibody decorates the *wBm* in cyan, scale bar = 4 microns, (i) Hypodermis, (ii)(iii) distal and proximal Proliferative Zone -PZ- areas, respectively, the red arrow points towards the DTC-, (iv)(v) distal and proximal Meiotic Zone -MZ- areas, respectively. The positions of the confocal view along the ovary are marked by colored boxes; red, DTC; blue, PZ; green and yellow, MZ. Grey arrows and dotted lines indicate the cutting points during the dissection. (D) Ongoing dissection in a glass spot plate. The gut fragment is discarded, the two ovaries are pulled out of the body wall, the PZs are cut first, and the second cuts isolate the MZs as indicated by grey arrowheads. Uteri are discarded (cf [30]). Scale Bar = 1mm.

We performed dual-RNAseq analyses on *B*. *malayi* tissues where the *Wolbachia* are present, the female germline and the hypodermis. In filarial nematodes, a pair of folded ovaries is located in the posterior of the female (Fig 1A and 1B). Each ovary is a tube containing a germline organized as a syncytium, starting with a distal part enriched in germline stem cells, referring here to syncytial nuclei whose daughters or progenitor cells expand through a transit amplification phase in the proliferative zone (PZ), and ending with the completion of the pre-meiotic S phase. Next, meiotic differentiation gives rise to cellularized oocytes loaded with mRNAs crucial for early embryogenesis, progressing up to the diakinesis stage of meiotic prophase I in the proximal part of the meiotic zone (MZ), before entering the oviduct. Although the somatic part of the ovary is made of a thin epithelium of sheath cells, we considered its contribution to the ovarian transcriptome to be negligible compared to the germline.

The distal germinal stem cells (GSCs) show the highest titer of endosymbionts compared to the following germline developmental stages [30]. The *Wolbachia* titer, however, remains high enough at any stage, allowing a dual RNAseq approach (Fig 1C). We dissected ovaries and physically separated ovarian fragments corresponding to the mitotic proliferation zone, that occupies the first millimeter of the ovary, from the meiotic differentiation zone (Fig 1D) [30]. Once the ovaries were dissected and the intestine discarded, we kept the body wall (BW), made of the hypodermis, the attached underlying muscles, as well as the thin dorsal and ventral nerve chords considered to be negligible in terms of gene expression. Because the *Wolbachia* are only present in the hypodermis, the BW fragment contains only hypodermal *Wolbachia*. However, the BW sample used in this RNAseq study reflects the *Brugia malayi* female gene expression in both muscles and hypodermal tissues.

A total of 50 females per biological replicate (100 tissue fragments per PZ and MZ, and 50 per BW) were pooled, and used to generate dual-RNAseq libraries (cf. Methods). Three biological replicates were prepared for each tissue. The sequencing of these nine libraries yielded 19.9 to 39.7 million reads. 1.2% to 12% of them aligned with the *wBm* genome, 1.1% to 8.9% with the *B*. *malayi* mitochondrial genome, and 39.8% to 81% with the *B*. *malayi* genome (Table 1). This last variability is due to the low number of total paired reads obtained specifically for the BW samples, from 39.8 to 53.9%, while the PZ and MZ samples yielded from 73.2 to 81% of reads. We noticed that a majority of *de novo* contigs assembled in the BW libraries are made of low complexity sequences and may, to some extent, have led to removal of reads from the alignments. It is also possible that the physicochemical properties of the bodywall make this sample more susceptible to RNA degradation. A significant difference is also observed for the *wBm* reads, many more of which mapped to the *wBm* genome in PZ (8.6 to 12%) compared to MZ (1.2–2.3%), reflecting the observed gradient of the *wBm* titer in the ovary (Fig 1C), with the highest concentration in the distal PZ. Throughout this study, for both the endosymbiont and *B*. *malayi* host gene expression analyses, genes with a log2 fold change (FC) >2, and FDR<0.01 were identified as differentially expressed, and their subsequent gene expression levels were validated by RT-PCR and qRT-PCR assays (cf. Methods and S1 Fig).

**Table 1:** Dual RNAseq sequencing summary. Total paired-end reads sequenced and aligned on the *Wolbachia wBm* genome, on the *B*. *malayi* mitochondrial genome and on the *B*. *malayi* genome in each biological replicate (1 to 3) for each sample (PZ: proliferative zone; MZ: meiotic zone; BW: body wall), and expressed as a total number of paired reads and as a percentage of paired reads per replicate.

	PZ-1	PZ-2	PZ-3	MZ-1	MZ-2	MZ-3	BW-1	BW-2	BW-3
Total Pairs	32 390 689	27 452 055	28 882 220	19 999 591	24 151 891	26 235 956	39 722 513	34 133 170	37 675 118
*wBm* Pairs	2 777 561	2 868 138	3 453 949	456 194	561 466	307 024	3 320 924	1 381 157	3 562 341
	8,6%	10,4%	12,0%	2,3%	2,3%	1,2%	8,4%	4,0%	9,5%
*Bm* Mitochondrion Pairs	843 307	640 482	903 966	247 906	275 804	344 490	3 301 452	2 281 642	3 370 610
	2,6%	2,3%	3,1%	1,2%	1,1%	1,3%	8,3%	6,7%	8,9%
*Bm* Pairs	24 003 870	20 679 992	21 135 828	16 001 916	19 351 819	21 247 243	15 815 815	14 073 255	20 321 115
	74,1%	75,3%	73,2%	80,0%	80,1%	81,0%	39,8%	41,2%	53,9%
Gerbil Pairs	116 622	127 667	109 732	92 997	109 174	84 115	1 137 172	474 221	359 831
	0,4%	0,5%	0,4%	0,5%	0,5%	0,3%	2,9%	1,4%	1,0%
% Total Mapped Pairs	85,6%	88,6%	88,6%	84,0%	84,0%	83,8%	59,4%	53,4%	73,3%

### *wBm* gene expression levels are largely similar between tissues and suggest high division rate in the soma, despite a harsh hypodermal environment

**Fig 2:**
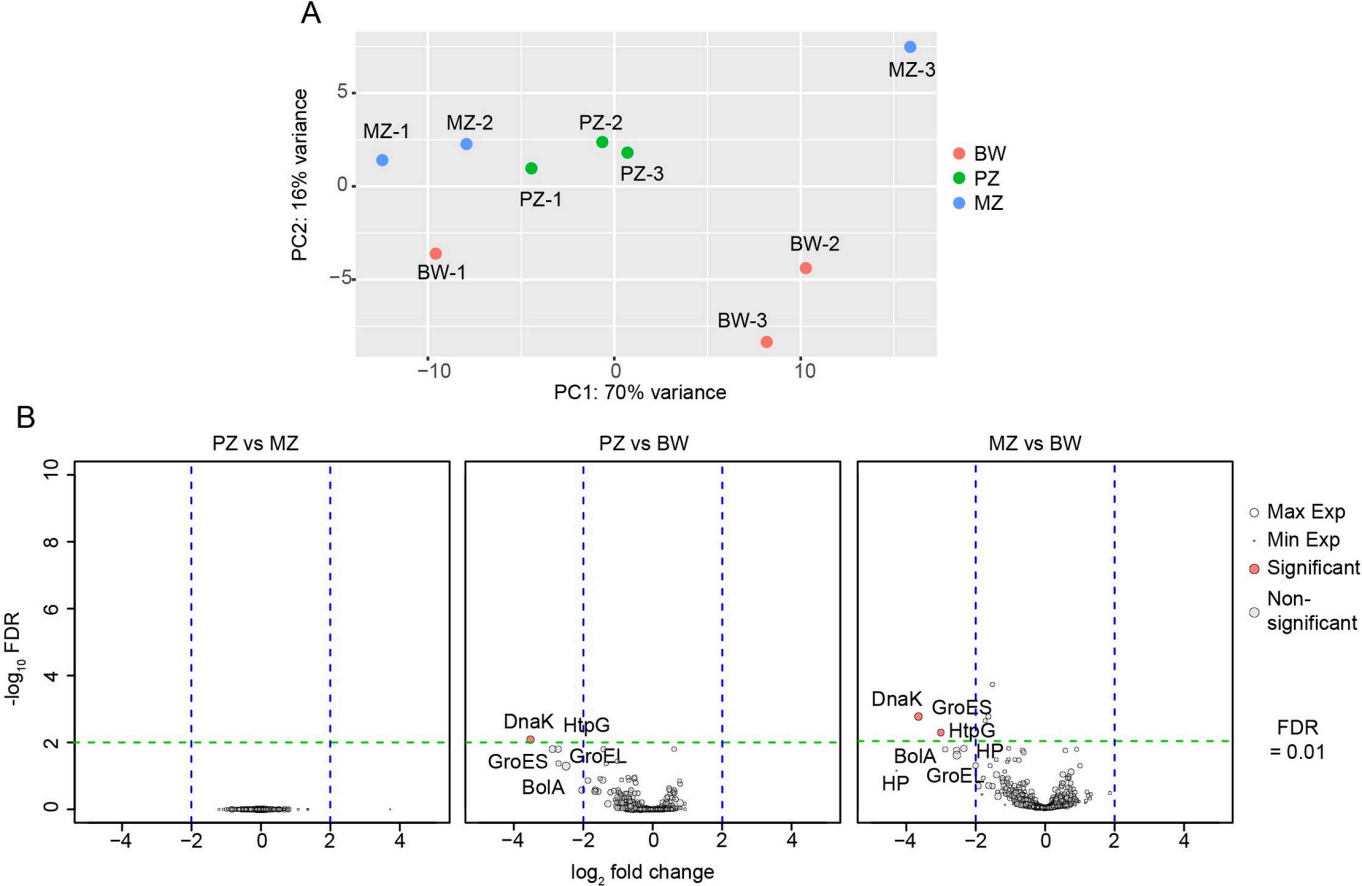
*wBm* gene expression levels in the *B*. *malayi* female germline and soma. (A) Principal component analysis of the 250 most variable *Wolbachia wBm* genes showing the distribution of the tissue-specific replicates. PZ: proliferative zone (green); MZ: meiotic zone (blue); BW: body wall (red). (B) Volcano plot representations of the *wBm* gene expression level variations between samples. PZ: proliferative zone; MZ: meiotic zone; BW: body wall. Y axis: -log_10_ (FDR); X axis: log_2_ (fold change). The dot size reflects the gene expression level, and red dots are genes whose expression level is considered to be variable between samples upon the following criteria: |log_2_(fold change)| > 2 with a FDR < 0.01.

We first evaluated the consistency between replicates regarding the *wBm* gene expression using a principal component analysis -PCA-, performed on the 250 most variable *wBm* genes (ranked on the basis of their respective variance after variance stabilizing transformation). The PCA showed that two replicates, MZ-3 (1 out of 3 for MZ) and BW-1 (1 out of 3 for BW) did not cluster well with their cognate replicates (Fig 2A). They were nonetheless considered as the result of a biological variation among the samples and were included in the analysis.

The data set revealed that except for one gene (WBM_RS03020) coding for a hypothetical protein whose expression is not detected in MZ, the 1006 *wBm* predicted CDSs are all expressed in the three different samples, defined as having at least 4 cumulative reads per independent biological replicate). A pairwise comparison of the PZ and the MZ segments shows no differentially expressed *wBm* genes within the different oogenesis stages (Fig 2B). Similarly, comparison of the *wBm* gene expression levels between the germline and the somatic BW samples identified very few differentially regulated genes, of which only one gene is upregulated in the BW compared to the PZ and two are upregulated in the BW compared to the MZ. The *wBm* genes significantly upregulated in the BW encode proteins described to play a role in stress response and chaperone complexes *(dnaK and groE*S*)*. It is noteworthy that other stress genes or chaperones *(bolA*, *grpE*, *htpG*, *hslV*, *groEL*) are also upregulated, although none of them in a significant manner (FDR>0.01, Fig 2B and Table 2). Since *wBm* possesses a functional type IV secretion system (T4SS), we identified the expression levels of the identified putative *wBm* effectors [31]. We did not find any significant variations between the different samples, suggesting that the cellular environments in the adult female do not influence the expression of the putative *wBm* T4SS effectors. Additionally, we ranked all putative effector genes based on their expression levels (Table 3). More generally, 99.8% of *wBm* genes show the same expression level—|log2(foldchange)|<2 and FDR>0.01 across all samples. Genes predominantly active in PZ and MZ also comprise genes involved in stress response or chaperone complexes. In all samples, the top expressed genes also include factors involved in transcription (i.e. the DNA-directed RNA polymerase subunit beta/beta' WBM_RS05535) and protein synthesis (i.e. the 30S ribosomal protein S1 -WBM_RS00350-, the 50S ribosomal protein L27 -WBM_RS04270-, or genes coding for elongation factors such as Tu -WBM_RS02015, WBM_RS03935-, or the elongation factor G -WBM_RS02020-). In addition, genes involved in ATP synthesis (F0F1 ATP synthase subunits alpha and beta, -WBM_RS01870, WBM_RS04200-) and genes involved in the electron transport chain also show a high expression in the three different samples. Similarly, the expression level of enzymes involved in pyruvate catabolism also rank high, including pyruvate phosphate dikinase (WBM_RS01250) and pyruvate dehydrogenase complex dihydrolipoamide acetyltransferase (WBM_RS04585), suggesting a high energy production through the TCA cycle or the malate dismutation pathway (S1 Table). Together, these results point towards similar *wBm* gene expression profiles in soma and germline, likely reflecting similar bacterial growth rates. They however highlight specific properties of the hypodermal chords, leading to the upregulation of *Wolbachia* genes involved in stress response.

**Table 2:** Summary of differentially expressed *wBm* genes. Expression level -normalized counts- and fold change of *wBm* genes for PZ, MZ and BW samples, considered differentially expressed if |log_2_(fold change)| > 2 and the FDR < 0.01 -in bold -, HP: hypothetical protein.

Current locus-tag	Former locus-tag	Expression level			Log2 fold change			Gene name
		PZ	MZ	BW	PZ vs MZ	PZ vs BW	MZ vs BW	
WBM_RS02060	Wbm0350	12040,8	11690,1	68129,9	0	-2,5	-2,5	GroEL
WBM_RS02930	Wbm0495	4603,2	4233,2	53051,5	0,1	**-3,5**	**-3,6**	**DnaK**
WBM_RS02055	Wbm0349	2610,8	2411,2	19349,8	0,1	-2,9	**-3**	**GroES**
WBM_RS00845	Wbm0138	1807,7	2044,6	11975,9	-0,2	-2,7	-2,6	HtpG
WBM_RS02355	Wbm0399	3280,4	2039,7	10352,4	0,7	-1,7	-2,3	HP
WBM_RS04440	Wbm0722	1946,4	2106,6	8037,7	-0,1	-2	-1,9	HslV
WBM_RS03155	Wbm0533	1013,9	926,4	3712,6	0,1	-1,9	-2	GrpE
WBM_RS02925	Wbm0494	441,7	395,2	2899,0	0,2	-2,7	-2,9	BolA
WBM_RS03020	Wbm0510	15,1	1,6	22,5	3,7	-0,6	-4,3	HP

**Table 3:** Expression levels of *wBm* putative effector genes screened by *Carpinone et al 2018*. Expression levels, with fold changes and FDR of putative *wBm* effectors in PZ, MZ and BW. High to low gene expressions are visualized with a red to blue coloured scale. Putative effectors according to *Carpinone et al 2018*, with the following authors' criteria: S, detected in *wBm* secretome; *Bm*, detected in dissected *Brugia malayi*; E, predicted eukaryotic-like protein motifs; 4, co-regulated with *wBm* Type IV secretion apparatus or similar to predicted Type IV effectors from related organisms; U, unique to *Wolbachia*.

Rank	Former locus-tag (*Carpinone et al*., *2018*)	Current locus-tag	Predicted function (*Carpinone et al 2018*)	Reason (*Carpinone et al*., *2018*)	Expression PZ	Expression MZ	Expression BW	PZ vs MZ		PZ vs BW		PO vs BW	
								Log2 fold change	FDR	Log2 fold change	FDR	Log2 fold change	FDR
13	WBM0181	WBM_RS01075	ATP-binding chaperone	Bm, 4	4591,5	4881,4	6076,1	-0,09	0,9995	-0,40	0,9936	-0,32	0,8812
26	WBM0432	WBM_RS02570	WSP family	S, Bm	3088,1	2152,8	3875,6	0,52	0,9995	-0,33	0,9936	-0,85	0,2998
15	WBM0209	WBM_RS01250	pyruvate phosphate dikinase	Bm	2944,9	3003,1	2382,6	-0,03	0,9995	0,31	0,9900	0,33	0,7591
7	WBM0076	WBM_RS00455	WAS(p) family protein, proline-rich	E	2718,3	2993,1	3492,1	-0,14	0,9995	-0,36	0,9936	-0,22	0,9350
14	WBM0193	WBM_RS05020	hypothetical, tropomyosin-like	Bm, E	2628,9	2828,1	3613,8	-0,11	0,9995	-0,46	0,8067	-0,35	0,7591
41	WBM0749	WBM_RS04600	hypothetical protein	Bm,4	2271,8	2377,6	1934,1	-0,07	0,9995	0,23	0,9757	0,30	0,6122
29	WBM0484	WBM_RS02870	hypothetical protein	S	2166,7	2444,6	2302,2	-0,17	0,9995	-0,09	0,9936	0,09	0,9601
10	WBM0152	WBM_RS00910	PAL-like	S, E	1773,8	1641,4	2095,4	0,11	0,9995	-0,24	0,9936	-0,35	0,8988
23	WBM0384	WBM_RS02275	ankyrin repeat protein, metalloprotease	E	1639,5	1662,6	1418,5	-0,02	0,9995	0,21	0,9936	0,23	0,8275
36	WBM0672	WBM_RS04090	hypothetical protein	Bm, U	1355,7	1237,8	2069,9	0,13	0,9995	-0,61	0,9099	-0,74	0,5639
25	WBM0430	WBM_RS02560	invasion associated protein B	S	1283,2	1261,7	2129,2	0,03	0,9995	-0,73	0,9099	-0,76	0,6729
32	WBM0582	WBM_RS03480	ankyrin repeat protein	S	1268,8	1401,3	1842,3	-0,14	0,9995	-0,54	0,1208	-0,39	0,3281
2	WBM0032	WBM_RS00195	tRNA modification GTPase	4	1196,3	1278,3	1296,8	-0,10	0,9995	-0,11	0,9936	-0,01	0,9739
22	WBM0307	WBM_RS01830	cytochrome C oxidase subunit	4	1179,0	917,3	1253,8	0,36	0,9995	-0,09	0,9936	-0,45	0,4983
38	WBM0711	WBM_RS04370	DNA recombination *rmuC*-like	S, Bm, 4	1144,5	1254,3	1305,9	-0,13	0,9995	-0,19	0,9936	-0,06	0,9712
43	WBM0752	WBM_RS04615	hypothetical protein	4	1009,0	1058,0	961,0	-0,07	0,9995	0,07	0,9947	0,14	0,9712
17	WBM0222	WBM_RS01325	preprotein translocase YajC	4	835,5	831,4	1290,6	0,01	0,9995	-0,63	0,2677	-0,64	0,1807
34	WBM0666	WBM_RS04035	dihydrolipoamide acetyltransferase	4	832,3	779,8	1198,8	0,09	0,9995	-0,53	0,1120	-0,62	0,0216
37	WBM0709	WBM_RS04335	coprophyrinogen III oxidase	4	773,9	767,6	803,7	0,01	0,9995	-0,05	0,9936	-0,06	0,9727
18	WBM0277	WBM_RS01625	calcineurin-like phopshodiesterase	4	751,0	863,2	639,4	-0,20	0,9995	0,23	0,9936	0,44	0,4376
20	WBM0287	WBM_RS01680	ankyrin repeat protein	E	700,3	791,8	1047,1	-0,18	0,9995	-0,58	0,9099	-0,40	0,8275
8	WBM0100	WBM_RS00600	Wolbachia Surface Protein (WSP) family	S	610,3	681,0	540,5	-0,17	0,9995	0,17	0,9936	0,34	0,8203
6	WBM0070	WBM_RS00425	Mg2+/Co2+ transporter	4	609,8	676,6	588,5	-0,15	0,9995	0,05	0,9936	0,20	0,8811
39	WBM0736	WBM_RS04525	secreted FK506 binding protein-like	Bm	538,4	581,6	579,7	-0,11	0,9995	-0,10	0,9936	0,01	0,9913
27	WBM0452	WBM_RS02690	SAM-dependent methyltransferase	E, 4	533,1	628,6	528,6	-0,24	0,9995	0,01	0,9986	0,25	0,8988
35	WBM0671	WBM_RS04085	DNA uptake lipoprotein	/	514,1	455,4	579,3	0,18	0,9995	-0,17	0,9936	-0,35	0,6933
21	WBM0290	WBM_RS01695	D-alanyl D-alanine carboxypeptidase	S	505,4	536,5	531,1	-0,09	0,9995	-0,07	0,9936	0,02	0,9727
19	WBM0284	WBM_RS01665	WSP family	4	502,4	296,9	630,3	0,77	0,9995	-0,33	0,9936	-1,10	0,3935
28	WBM0482	WBM_RS02855	hypothetical, tropomyosin-like	Bm, E	485,2	470,7	525,5	0,04	0,9995	-0,11	0,9936	-0,15	0,9450
11	WBM0164	WBM_RS00975	hypothetical, tropomyosin-like	E	417,2	517,5	399,9	-0,32	0,9995	0,06	0,9947	0,38	0,8502
42	WBM0751	WBM_RS04610	hypothetical protein	4	378,7	361,8	740,8	0,07	0,9995	-0,97	0,6277	-1,04	0,3935
9	WBM0114	WBM_RS00705	peptide deformylase	4	374,6	356,6	423,2	0,08	0,9995	-0,17	0,9936	-0,26	0,8450
45	WBM0792	WBM_RS04855	hypothetical protein	E	367,7	388,6	316,6	-0,07	0,9995	0,21	0,9936	0,28	0,8369
5	WBM0064	WBM_RS00385	FAD-dependent oxidoreductase	/	362,5	344,3	479,7	0,07	0,9995	-0,40	0,8311	-0,47	0,5025
16	WBM0213	WBM_RS01280	hypothetical protein	E, U	340,6	393,3	298,4	-0,21	0,9995	0,19	0,9936	0,40	0,5690
44	WBM0791	WBM_RS04850	N6-adenine methylase	/	327,8	371,9	332,5	-0,18	0,9995	-0,02	0,9962	0,17	0,8848
1	WBM0014	WBM_RS00085	ABC transporter	4	301,0	293,6	272,7	0,04	0,9995	0,15	0,9936	0,12	0,9601
4	WBM0057	WBM_RS00340	ZapA superfamily, hypothetical	/	295,5	354,0	322,4	-0,25	0,9995	-0,13	0,9936	0,12	0,9712
3	WBM0044	WBM_RS00260	amino acid transporter	Bm	279,8	289,0	239,0	-0,06	0,9995	0,22	0,9936	0,28	0,8917
31	WBM0506	WBM_RS03000	outer membrane protein	E	244,7	227,1	258,1	0,09	0,9995	-0,08	0,9936	-0,16	0,9476
30	WBM0491	WBM_RS02910	cytochrome C oxidase assembly protein	S	176,5	159,1	330,4	0,16	0,9995	-0,90	0,1324	-1,06	0,0356
33	WBM0665	WBM_RS04030	ankyrin repeat protein	4	175,6	209,6	280,5	-0,27	0,9995	-0,68	0,6013	-0,41	0,7800
12	WBM0165	WBM_RS00980	hypothetical protein	U	129,5	190,7	185,2	-0,57	0,9995	-0,52	0,9936	0,04	0,9739
40	WBM0748	WBM_RS04595	hypothetical protein	4	106,1	102,0	129,2	0,02	0,9995	-0,29	0,9936	-0,31	0,9194
24	WBM0394	WBM_RS02325	ankyrin repeat protein	E	100,6	91,9	119,4	0,09	0,9995	-0,25	0,9936	-0,35	0,9040
46	WBM0772	removed	hypothetical protein	Bm, E	/	/	/	/	/	/	/	/	/
47	WBM0447	removed	ankyrin repeat protein	S, Bm, E	/	/	/	/	/	/	/	/	/

### *B*. *malayi* gene expression levels during oogenesis and in the body wall

**Fig 3:**
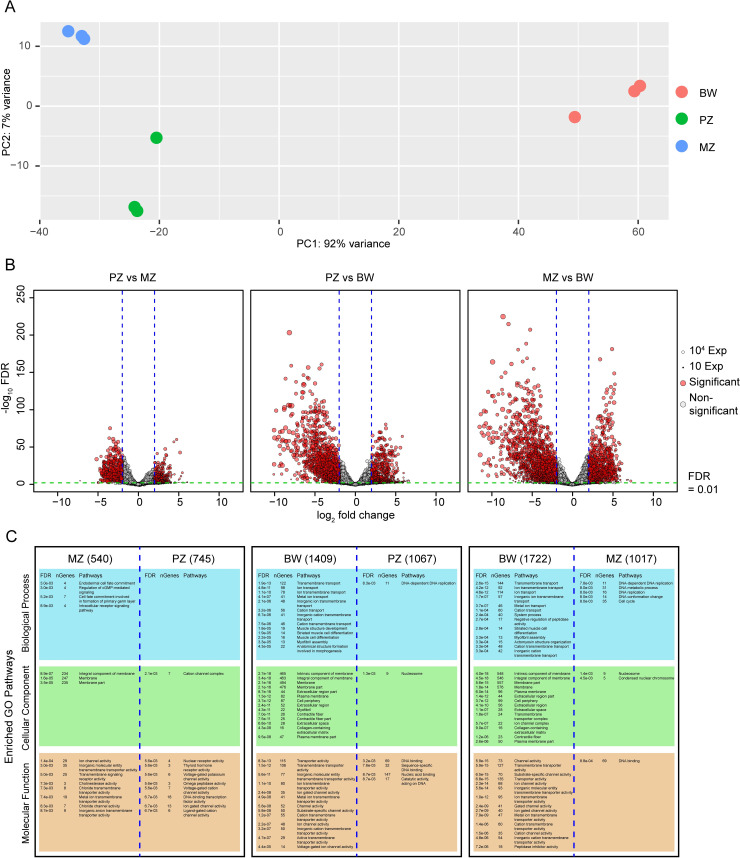
*B*. *malayi* gene expression analyses. (A) PCA of the 2000 most variable *B*. *malayi* genes in 3 biological replicates for each analyzed tissue, PZ (green); MZ (blue); BW (red). (B) Volcano plots representing differences in gene expression levels between PZ, MZ and BW samples. X axis: log_2_(fold change); Y axis: -log_10_ (FDR). Dot size corresponds to gene expression level. Red dots are genes significantly regulated when |log_2_(fold change)| > 2 and the FDR < 0.01. (C) GO enrichment pathways for each compared tissue for the 3 principal GO categories: biological process (blue); cellular component (green); and molecular function (orange). nGenes: number of genes identified in each category. Numbers in brackets correspond to the number of genes identified as regulated in each tissue for each comparison.

The PCA performed on the 2000 most variable *B*. *malayi* genes shows the replicates for each of the BW, PZ, and MZ samples to cluster together (Fig 3A). Among the 11,002 *B*. *malayi* protein coding genes, only 121 (1.1%), 355 (3.2%) and 545 (4.9%) show no expression in the PZ, MZ, and BW samples respectively (i.e. a minimum of four cumulative reads across the independent biological replicates).

A comparison of the genes expressed during mitotic proliferation and meiotic differentiation in the ovary (in PZ and MZ samples respectively) shows that 745 genes are upregulated in PZ, with an enrichment of Gene Ontology -GO- terms associated with ion channel activity, receptor activity and transcription factor activity (using iDEP.9.1 -integrated Differential Expression and Pathway analysis-, see Methods). During meiotic differentiation, 540 genes are upregulated in the MZ samples with an enrichment of GO terms associated with cell fate, signaling pathway, membrane component and transmembrane transporter activity. The comparison between genes expressed in the BW versus those expressed in the two ovarian regions (PZ and MZ) reveals the highest number of differentially expressed genes, with 1409 and 1722 genes being up regulated in BW compared to PZ and MZ, respectively. For both sets of differentially-expressed genes, we find enrichment of GO terms associated with transmembrane transport, muscle development, membrane components and ion channel activity. In the ovary, 1067 and 1017 genes are upregulated in PZ and MZ, respectively, compared to BW with an enrichment of GO terms associated with DNA processes, cell cycle, and DNA binding in both gene sets (Fig 3B and 3C; S2 Table).

### Comparison with *C*. *elegans* germline transcriptomics reveals a poor conservation of *B*. *malayi* genes involved in oogenesis, especially in the proliferative zone

In an attempt to better examine the genes involved in adult female oogenesis in *B*. *malayi*, we compared the germline transcriptome of *B*. *malayi* to that of *C*. *elegans*, whose germline has been extensively studied. *C*. *elegans* is hermaphrodite, the same gonad producing sperm prior to oocytes, due to a temporally-regulated germline genetic programme [33]. Genetic manipulation in this experimental model has allowed the generation of mutants that only make either sperm or oocytes. We used the most recent transcriptomic dataset generated from spermatogenic and oogenic *C*. *elegans* gonads to assess the potential evolutionary conserved mechanisms in the making of a female germline between these species [34].

**Fig 4:**
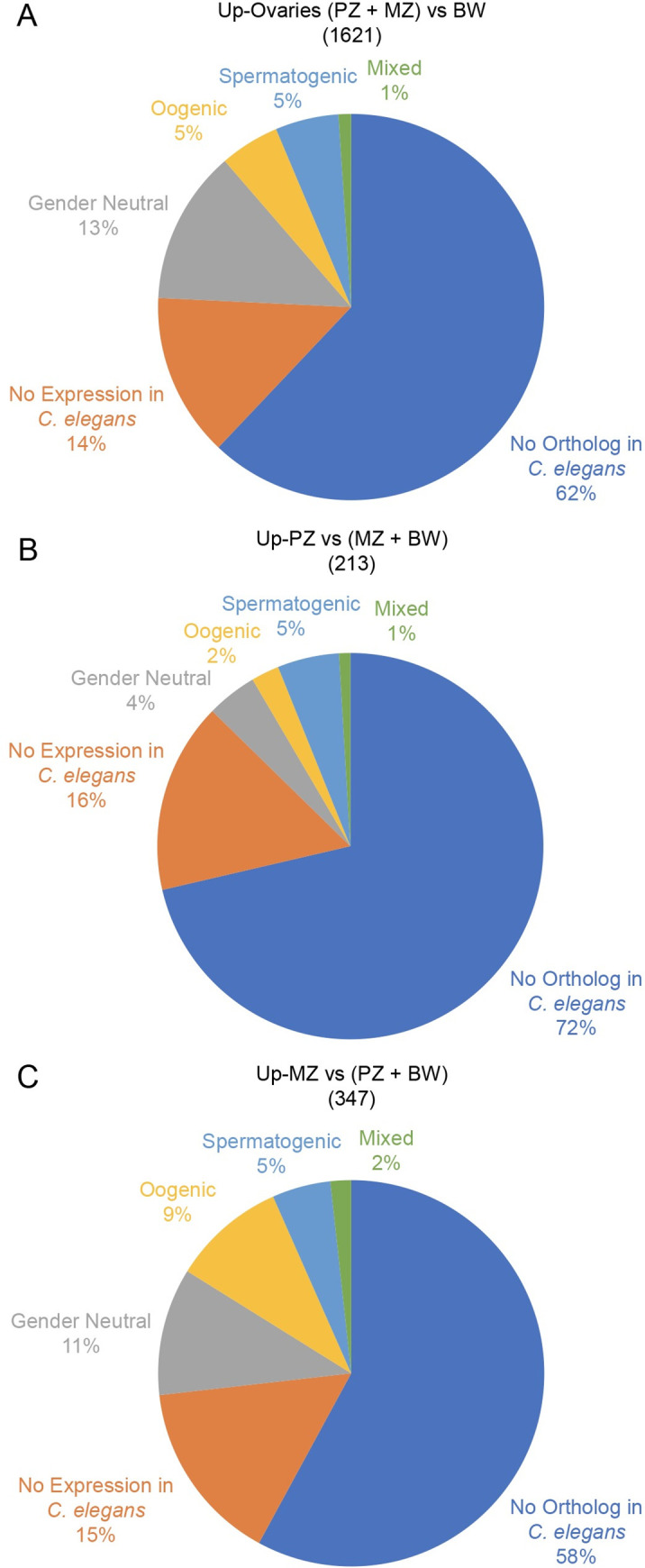
*B*. *malayi* female germline genes presenting *C*. *elegans* orthologs. Pie charts of genes specifically expressed in *B*. *malayi* ovaries (PZ+MZ), PZ and MZ, classified according to their orthology with genes expressed in the *C*. *elegans* gonad. *C*. *elegans* genes were considered as spermatogenic when upregulated in *fem-3(gf)* spermatogenic gonads (light blue), oogenic when upregulated in *fog-2* oogenic gonads (yellow) or gender neutral when no differential expression was observed between mutant gonads (gray), based on a two-fold difference in abundance and a FDR<0.01[34]. Expressed *B*. *malayi* genes having *C*. *elegans* orthologs but not expressed in the *C*. *elegans* gonad are coloured in orange. Expressed *B*. *malayi* genes without *C*. *elegans* orthologs are coloured in dark blue. Expressed genes with >2 *C*. *elegans* orthologs belonging to at least 2 different *C*. *elegans* expression status are called mixed, and coloured in green.

Among the 11002 protein-coding genes of *B*. *malayi*, 6048 genes -55%- share orthologs with *C*. *elegans*. We compared the 1621 *B*. *malayi* genes specifically upregulated in the female germline (PZ+MZ versus BW), to their orthologs expressed in the *C*. *elegans* gonad (Fig 4A). We found that only 5% of genes specifically upregulated in the *B*. *malayi* female germline have orthologs described in *C*. *elegans* as "oogenic", with a similar proportion described as "spermatogenic" genes. 13% of the *B*. *malayi* ovarian genes have orthologs intrinsic to the *C*. *elegans* germline and therefore are expressed in both oogenic and spermatogenic *C*. *elegans* germlines -i.e. *gender neutral*-. Furthermore, 14% of genes expressed in the *B*. *malayi* female germline have no orthologs in the *C*. *elegans* gonads. Additionally, alignment tools from the Ensembl pipeline (cf. Methods) fail to detect *C*. *elegans* orthologs for 62% of the genes specifically expressed in the filarial ovary (S3 Table).

To look at the resolution of developmental phases during oogenesis, we first focused on the *B*. *malayi* transcriptome of the germline mitotic proliferation zone PZ. The 213 specific genes involved in the earliest steps of germline development -upregulated in PZ compared to MZ and BW- appear even more evolutionarily distant from those expressed in *C*. *elegans*, since only 2% have oogenic orthologs, and 72% of them do not present any orthology with *C*. *elegans* genes (Fig 4B). If the regulation of the mitotic proliferation and the meiotic switch are comparable mechanisms to that of *C*. *elegans*, the transcripts found upregulated in PZ should reflect specific cell fate and behavioral traits in the distal ovary, more specifically involving germline stem cell fate, proliferation, and accumulation of transcripts later involved in meiosis. We found among the 2% oogenic orthologous genes having a described function in *C*. *elegans*: *bed-3* (WBGene00223856 ortholog in *B*. *malayi*); *ztf-1* (WBGene00224529); *suro-1* (WBGene00225602); and *cht-1* (WBGene00228562). Although found to be upregulated in the oogenic *C*. *elegans* germline, none of these genes has a described function in the germline. Similarly, there are no known germline functions for the spermatogenic orthologs. We then searched for the gender-neutral orthologs in PZ, found upregulated in both oogenic and spermatogenic germlines in *C*. *elegans*. Among the 11 gender-neutral genes, we found 4 orthologs with a described function in the germline to be expressed in PZ: 2 orthologs of *cdk-1* involved in cell cycle and proliferation [35] (WBGene00230408; WBGene00230411), *msh-5* involved in chiasma assembly [36] (WBGene00223490), and *prom-1*, coding for an F-box protein described to allow meiotic progression and chromosome pairing [37] (WBGene00229468). Together these results indicate that the mechanisms controlling stem cell fate and mitotic proliferation, as well as progression into meiosis in the *B*. *malayi* adult female germline rely on the expression of genes dramatically different or divergent compared to *C*. *elegans* (S3 Table).

We similarly examined 347 genes whose transcripts are upregulated during the meiotic prophase I and up to diakinesis occurring in the proximal MZ in the ovary -upregulated in MZ compared to PZ and BW-. Specifically, 9% have oogenic, 5% spermatogenic, and 11% gender neutral orthologs (Fig 4C). The conserved 31 oogenic gene orthologs expressed in MZ comprise genes likely to be involved in meiosis: *efl-3* (WBGene00224495) a E2F protein that regulates cell death; *gld-1* (WBGene00226227) involved in oocyte differentiation [38]; and the cyclin *cyd-1* (WBGene00226476). Additionally, we find genes coding for maternal transcripts loading the oocyte and playing key roles in early *C*. *elegans* embryogenesis, such as *mom-5* (WBGene00226206) a Wnt pathway component [39]; *pal-1* (WBGene00222924) coding for a homeodomain protein involved in blastomere identity and maintenance in the *C*. *elegans* embryo [40]. We also find genes with roles in early embryonic polarity such as *par-3* (WBGene00224636) [41] or the *lit-1* kinase (WBGene00226476, 42] and in cell cycle with the cyclin *cyb-3* (WBGene00225880) controlling division in the early *C*. *elegans* embryo [43]. The 18 spermatogenic gene orthologs found in MZ have no known functions described in the germline. Among the 44 gender neutral orthologs we find *fcp-1* (WBGene00224135) a phosphatase that regulates meiotic maturation [42]; the cyclin *cya-1* (WBGene00227740) involved in meiotic cell cycle; and the E3 ligase *cul-2* (WBGene00231090) which plays a role in meiosis and early embryonic polarity [44]. The other orthologs do not have described functions in the germline. In summary, genes expressed during meiotic prophase I in the *B*. *malayi* MZ share more germline orthologs with *C*. *elegans*, especially oogenic and gender neutral specific genes compared to the PZ, suggesting that meiosis and early embryogenesis may rely on more conserved evolutionary mechanisms. Nevertheless, 58% of the genes expressed in MZ share no orthologs with *C*. *elegans* (S3 Table).

### Development and maintenance of the distal germline -PZ- rely on the highest number of filarial specific genes of the Onchocercidae family

The majority of genes expressed in the *B*. *malayi* germline or body wall do not have *C*. *elegans* orthologs. By using a comparative genomic approach, we sought to assess the phylogenetic origin of the expressed genes in the ovary and BW samples to potentially detect orthologous genes present outside *C*. *elegans* and to determine which genes may be the result of evolutionary innovation or adaptation within filarial nematodes.

**Fig 5:**
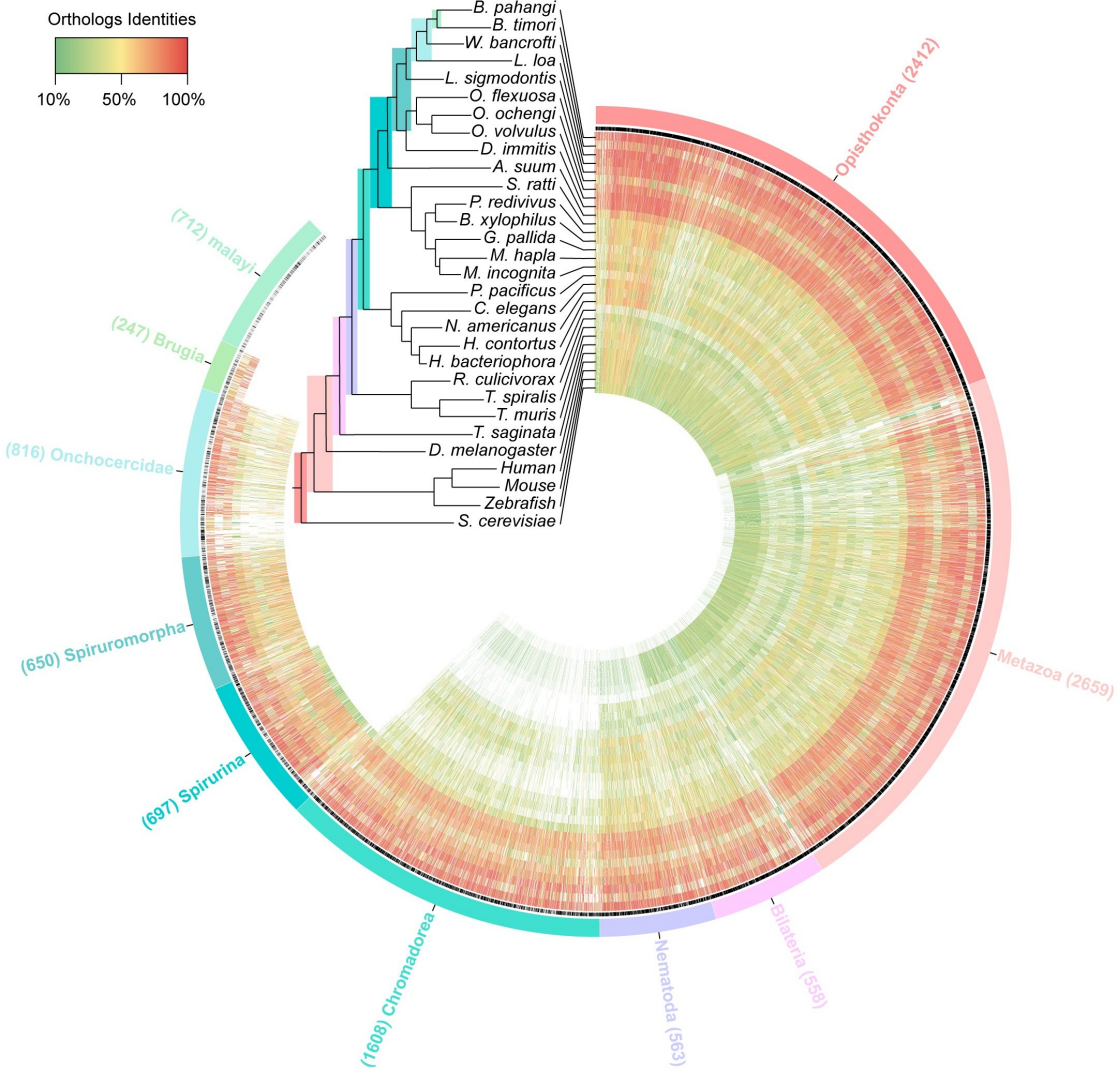
*B*. *malayi* orthology map. Circos plot representing 10,922 *B*. *malayi* genes and their orthologs with 24 Nematoda species, 1 Platyhelminth species and 5 outgroup species. Circles from outermost radius to innermost are: clade root origin of each *B*. *malayi* gene; presence or absence of GO functional annotation (black: presence; gray: absence); orthology identified for 30 species varies from red = 100% identity to green = 10% identity. The phylogenetic tree is adapted from [61], between the 30 following species: *Brugia pahangi*, *Brugia timori*, *Wuchereria bancrofti*, *Loa loa*, *Litomosoides sigmodontis*, *Onchocerca flexuosa*, *Onchocerca ochengi*, *Onchocerca volvulus*, *Dirofilaria immitis*, *Ascaris suum*, *Strongyloides ratti*, *Panagrellus redivivus*, *Bursaphelenchus xylophilus*, *Globodera pallida*, *Meloidogyne hapla*, *Meloidogyne incognita*, *Pristionchus pacificus*, *Haemonchus contortus*, *Heterorhabditis bacteriophora*, *Necator americanus*, *Caenorhabditis elegans*, *Romanomermis culicivorax*, *Trichinella spiralis and Trichuris muris*, *Taenia saginata*, *Drosophila melanogaster*, *Homo sapiens*, *Mus musculus*, *Danio rerio* and *Saccharomyces cerevisiae*.

To this end, we established an orthology map of the *B*. *malayi* genome, with a focus on genes specifically expressed in the ovary and the BW. The *B*. *malayi* genome contains 11002 protein-coding genes, of which 64 have no WormBase ID and 16 have a WormBase ID but do not exist in the WormBase Parasite Compara database (S4 Table). Orthology information was obtained for the remaining 10,922 *B*. *malayi* genes from the WormBase Parasite Compara database, through comparisons with 30 chosen species genomes (Fig 5). This orthology map of the *B*. *malayi* genome is divided in 10 different clade origin categories, from the Opisthokonta clade down to the species level “*malayi*". Of the 10,922 genes, 5,629 (51.5%) are shared by the Opisthokonta, Metazoa and Bilateria; 2,171 (19.9%) have a common origin within the Nematoda and Chromadorea; and 1,347 (12.3%) share a common origin with the Spirurina and Spiruromorpha. Importantly, 816 *B*. *malayi* genes have orthologs within the Onchocercidae only, a family of filarial nematodes in which some members share a symbiosis with *Wolbachia* [6]. At the genus level, 247 genes are specific to *Brugia*, and 712 genes appear unique to the *B*. *malayi* species.

**Fig 6:**
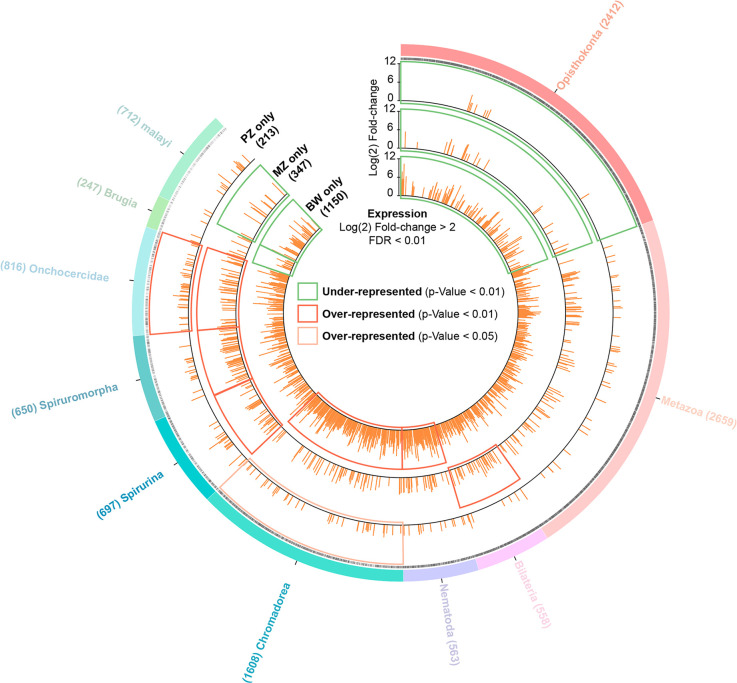
Distribution of *B*. *malayi* expressed genes at the taxonomic level. Circos plot representing 10,922 *B*. *malayi* genes with their level of fold change of expression. Circles from outermost radius to innermost: clade root origin of each *B*. *malayi* gene; presence or absence of GO functional annotation (black: presence; gray: absence); gene expression levels (orange bars) with a log_2_(fold change) > 2 and a FDR < 0.01 are considered specific for PZ (versus MZ+BW); MZ (vs PZ+BW); and for BW (vs PZ +MZ). Green squares highlight taxonomy levels that are under-represented in genes with a Fisher’s exact test p-value < 0.01; red squares highlight taxonomy levels over-represented in genes (Fisher’s exact test p-value < 0.01); and orange squares highlight taxonomy levels that are over-represented in genes with a Fisher’s exact test p-value < 0.05.

Using the gene subsets specifically up-regulated in one tissue compared to the two others, specifically the 213, 347 and 1150 genes up-regulated in PZ, MZ and BW, respectively, we performed an enrichment analysis, to establish the origin of the expressed genes for each tissue sample, among the 10 clade categories identified in the whole genome orthology map (Fig 6). The enrichment analysis indicates that the clades Nematoda (134 of 1150 genes) and Chromodorea (289 of 1150 genes) are enriched in genes up-regulated in BW. Bilateria (29 of 347 genes), then Spirurina (39 of 347 genes) to Onchocercidae (49 of 347 genes) clades are enriched in genes up-regulated in MZ. Finally, the analysis on PZ reveals that genes from the Onchocercidae clade are over-represented (with 46 genes out of 213 genes). This enrichment analysis suggests that genes expressed in the germline proliferative zone PZ are an important part of an evolutionary innovation limited to the Onchocercidae. However, the enrichment analysis on the MZ genes suggests that the meiotic program, and potentially early embryogenesis, seems instead to rely on genes more broadly shared in the Bilateralia, Nematoda and from the Spirunina to Onchocercidae clades. Taken together, this suggests that in *B*. *malayi*, the germline meiotic differentiation program is likely to rely on evolutionary conserved mechanisms in Bilateralia and among Nematoda, while the germline stem cell fate, the control of the germline proliferation and the regulation of the meiotic switch may require many evolutionary innovations and adaptations specific to filarial nematodes in the Onchocercidae, that may have contributed to the development of the symbiosis with *Wolbachia*, restricted to this family of nematodes within clade III.

## Discussion

In order to better understand the making of the germline in filarial species, and the potential adaptation and contributions of *Wolbachia* to somatic and germline tissues, we describe here the first tissue-specific transcriptomic profiling of the filarial nematode *Brugia malayi* and its associated *wBm* endosymbiont, that resolves the mitotic proliferation and meiotic differentiation stages in the adult female germline. Filarial nematodes are animal parasites constituting a major threat to terrestrial vertebrates including humans. Almost all filarial species that infect humans harbor *Wolbachia* [12], including *B*. *malayi*, an agent of human lymphatic filariasis. *B*. *malayi* shows a typical localization pattern for *Wolbachia*, present in the lateral hypodermal chords in adults as well as in the female germline to ensure its transmission to the progeny [9,10]. While we identified a few differentially regulated *Wolbachia* genes that indicate a stress response in hypodermal compared to germline bacterial populations, 99.8% of *wBm* genes share the same expression profile between tissues, including those coding for putative effectors. Genes related to active transcription and translation are among the most expressed genes, suggesting similar *wBm* growth in both tissues. These endosymbionts co-evolved with a host species whose germline, especially its mitotic proliferation phase, likely benefits from evolutionary innovations supporting the parasitic lifestyle, as suggested by the comparative analysis with *C*. *elegans* germline transcriptomics, and by the first orthology maps for the *B*. *malayi* genome and transcriptomic tissue-specific profiles.

A key factor in the parasitic lifestyle of filarial nematode species is to produce abundant progeny. *B*. *malayi* females release more than a thousand microfilariae every day in the human body, in order to increase chances of resuming their life cycle in a mosquito vector after a blood meal. To reach such figures, the female possesses a pair of ovaries whose PZs are composed of about 3,000 nuclei, multiplying in a transit amplification manner from a pool of GSCs. The most distal GSCs are quiescent, likely to provision the PZ with intact genetic information during the 5 to 8 years of female fertility [30]. Hence, the mechanisms of germline stem cell regulation and maintenance are of paramount importance in successfully perpetuating the species. This biological process has been extensively studied in the nematode *C*. *elegans*, as a paradigm of germline stem cell biology. However, 62% of genes specifically expressed in the filarial ovary have no orthologs in *C*. *elegans* at all, explained by considerable differences between these two species. In absence of paleontological data, molecular phylogeny estimates a ~350 My divergence between these species [32]. Although *C*. *elegans* possesses 20,470 CDSs, almost twice the number of genes coding for proteins in *B*. *malayi* -11,002-, and both species present similarly organized germlines, they belong to distant clades and have different lifestyles. The free-living *C*. *elegans* has a lifespan of several weeks with an optimal progeny of several hundred made from a pair of gonads of about 250 PZ nuclei. *B*. *malayi* produces millions of microfilariae during a lifespan close to a decade, in hypoxic conditions in the vertebrate host's lymphatics, likely impacting its metabolism [45]. A Notch-dependent mitotic proliferation of progenitor cells is controlled by the gonad distal tip cell -DTC- in *C*. *elegans*, that inhibits the translation of transcripts involved in meiosis in order to maintain the PZ [29]. We previously reported that in *B*. *malayi*, nuclei in PZ and MZ are clearly physically separated, and the DTC is dispensable for maintenance of the mitotic proliferation, pointing towards alternative somatic sources of an otherwise essential Notch ligand. Germline proliferation in *B*. *malayi* depends on both a Notch signaling pathway and the presence of *Wolbachia*. In absence of *wBm*, the PZ becomes reduced, and the GSCs lose their intrinsic properties, such as cycling and organization. This indicates a developmental symbiosis, with *wBm* endosymbionts being essential to the making of their host's germline [30].

The *Wolbachia* are extremely numerous in the ovary, with a density gradient increasing towards the distal tip and its GSCs (i.e. Fig 1C). This could explain the greater abundance of *Wolbachia* reads in the PZ samples compared to the MZ samples. Pair reads are nonetheless much more abundant for the host compared to the endosymbionts. This is due to technical limitations, such as the limited amount of biological material and its time-consuming collection, constraining our analyses to three biological replicates per sample for a dual RNAseq analysis, instead of purifying *wBm* from the tissues of interest to specifically enrich for *Wolbachia* reads. Although additional replicates would improve the fine resolution of the *wBm* gene expression profiles, our data are nonetheless likely to closely reflect the behavior of *wBm* in *B*. *malayi* raised in gerbils. *Wolbachia* must divide at a high rate to synchronize with the transit amplification pace during the germline mitotic proliferation, an activity *de facto* reflected in the transcriptomic analysis of *wBm* in PZ. Across the three samples, in both soma and germline, the most expressed *wBm* genes are involved in transcription and protein synthesis, but also in ATP production and pyruvate catabolism. This last observation is in agreement with pyruvate being at the host-endosymbiont metabolic interface since *wBm* are unable to perform glycolysis, and both pyruvate phosphate dikinase and pyruvate dehydrogenase complex dihydrolipoamide acetyltransferase are found to be upregulated by *in vitro* pyruvate supplementation [21,46,47]. The elevated expression of genes involved in ATP production also corroborates the results of previous transcriptomic and proteomic analyses of *wOo* in the filarial parasite *O*. *ochengi*, that suggests a nucleotide provisioning of the host in this symbiosis [23]. This high ATP production could also reflect the *wBm* energy demands of their own metabolism, since we previously did not identify a nucleotide depletion in *B*. *malayi* after *wBm* removal [30]. This observation could nonetheless result from compensatory mechanisms (i.e. an increased uptake from the vertebrate host, although *B*. *malayi* uses nucleotide salvage pathways rather than synthesizing nucleotides *de novo*) and we think the question remains open. Similar studies in *O*. *ochengi* [23] *and O*. *volvulus* [48] highlight a possible role of *wOo* and *wOv* in defense mechanisms modulating the vertebrate host immune response. The chaperones GroEL, GroES and DnaK are also among the most abundant *wOo* transcripts or proteins, suggesting a constitutive heat-shock/stress response, and GroEL is known in other bacteria as an immunomodulator acting as a ligand of the Toll-like receptors -2 & -4[23]. Most *Wolbachia* immunogenic products are likely to become exposed through the degradation of the microfilariae present in the mammalian body. Alternatively, some of these proteins may be secreted from the adult worm, but these *Wolbachia* chaperones have not been detected [49]. We identified the bacterial chaperones DnaK and GroES to be upregulated in the hypodermis, suggesting a metabolic activity that may in part account for defense mechanisms against an oxidative stress in order to maintain homeostasis. Hence the hypodermis may represent a harsh environment to which the endosymbionts must adapt accordingly. In addition, hypodermal chords are tissues with intense metabolic activities, filtering nutrients in a transcuticular manner from the host body fluids to provision the female germline and the demanding uteri, filled with developing embryos up to the microfilarial stage [50]. These upregulated *wBm* genes may be a consequence of a higher bacterial metabolic activity in rich conditions. This is in agreement with a previous stage-specific dual RNAseq analysis that reported *wBm* GroEL and GroES to be upregulated in adult *B*. *malayi* females, compared to males [51].

Nonetheless, the RNAseq analysis highlights similar expression levels for 99.8% of *wBm* genes across the different samples. Genes with the highest expression level have key roles in transcription and protein synthesis, suggesting similar *wBm* growth in hypodermis and germline. The syncytial hypodermal chords are however a finite space compared to the proliferative germline. Since we used young adult females in this analysis, it is not clear yet whether *wBm* endosymbionts were still actively colonizing the hypodermis or had already reached a maximal occupancy in this tissue. The latter hypothesis would imply a high turnover of endosymbionts regulated by the autophagy machinery. The induction of autophagy can in some instances influence the gene expression level of actors such as Atg8/LC3[52], as well as other key players of the autophagy pathway. The *B*. *malayi Atg8* ortholog is not upregulated in the hypodermis. However, neither transcript nor protein -i.e. ATG8 lipidation- levels at steady state provide sufficient information regarding induction of autophagy, and caution should be taken to draw conclusions without monitoring autophagy by flux measurements [52]. Therefore our experimental approach does not allow us to conclude on the role of autophagy in regulating hypodermal *wBm* populations, although this has been previously suggested [53].

Analyses of *B*. *malayi* orthology maps and comparison with the *C*. *elegans* germline transcriptomes revealed that a significant number of genes are specific to filarial nematodes in the process of meiosis, and the maintenance of the GSCs and their progenitors in PZ relies on even a greater number of genes absent from *C*. *elegans* (up to 72%). Enrichment analyses show that the genes found only in the Onchocercidae clade are over-represented in the transcriptomic profile of the PZ. Such orthology and enrichment analyses performed at the soma level, i.e. the bodywall, could be applied to other species in order to find macrofilaricidal drug target candidates, common or species-specific across filarial nematodes of biomedical importance. While 812 genes expressed in *B*. *malayi* are found in Onchocercidae only, caution should be taken regarding the number of genes specific at the species level "malayi" -712-, because the establishment of orthologies depends greatly on the quality of the currently available filarial genomes. Although some of these genes may have diverged in filarial nematodes to sufficiently preclude detection of orthologs in other species, it is very likely that many of these genes are evolutionary innovations that allow for species-specific parasitic lifestyles [2], i.e. enabling among other features a long-lasting fertility and an abundant progeny. Increasing evidence supports *de novo* genes arising from non-genic DNA sequences to create new functions [54], potentially supporting parasitism. The identification of these candidate genes will open the way to functional analyses using FISH and RNA silencing techniques to assess their roles in the germline and early embryonic development in *B*. *malayi*. However, the mechanism by which the *wBm* endosymbionts contribute to the worm's homeostasis or to this developmental symbiosis is still unclear. We hypothesize they modulate host gene expression in the filarial germline through secretion of T4SS effectors to maintain proper cell fates and the germline genetic program, making possible the production of viable oocytes. The transcriptomic approaches we established here will next allow us to address how *Wolbachia* impact the making of a filarial germline, at the GSCs, proliferation and meiotic levels.

## Methods

### Ethics statement

The study protocol regarding rearing of infected gerbils and *B*. *malayi* collection #03622.01 has been approved by the Ministère de l'éducation nationale, de l'enseignement supérieur et de la recherche, that granted a formal waiver of ethical approval.

### Parasite material

Live *B*. *malayi* females were sequentially collected from 3 infected gerbils (*Meriones unguiculatus*), at ages 3 to 5 months post-infection, and directly dissected. During the dissection process for RNAseq experiments, living worms were kept in culture medium (80% RPMI-1640, 10% decomplemented FBS, 10% MEM, 1% glucose, 25mM HEPES buffer, pH 7.4) at 37°C and 5% CO_2_.

### Tissue collection, RNA extraction and library preparation

Tissues used for RNA extractions were dissected from live adult worms. Dissections were performed under a binocular microscope equipped with a graduated eyepiece (SMZ1270; Nikon), in a RNAse-free environment by placing a female worm in ice-cold sterile PBS in a glass spot, using RNAseZap-cleaned tools (Applied Biosystems). Fragments corresponding to the ovarian proliferative (PZ) and meiotic (MZ) zones, as well as the body wall fragment (BW) were cut with tweezers from each dissected female. Specifically, distal gonads were extracted from the posterior body wall as described [30]. The PZ consists of the most distal 1000 μm of the ovary [30]. Two cuttings were performed with the tweezers, a first one to separate the PZ from the proximal ovary -MZ-, and a second one to discard the oviduct from the MZ. The tissue collection was performed in less than 5 minutes for each female, and the samples transferred with tweezers to RNAse-free eppendorf tubes kept on dry ice to instantly freeze the samples. Tissue fragments from 50 females were pooled together (a hundred fragments for PZ and MZ, and fifty for BW) to generate one biological replicate. Three biological replicates were prepared for each tissue by using a total of 150 females.

RNA extractions were performed using a Quick RNA MicroPrep kit (Zymo-Research). For all the samples, the RNA integrity and concentration were assessed on a RNA nano chip using a Bioanalyzer 2100 (Agilent Technologies) and a Qubit fluorometer (Invitrogen). All RNA-Seq libraries were prepared using the ScriptSeq Complete Gold kit (Epicenter)—Low Input from Epicentre/Illumina using ∼200 ng of total, DNase-I-treated RNA from samples as an input. Following the manufacturer’s instructions, *Wolbachia* and eukaryote host rRNAs were first removed using the Ribo-Zero Magnetic Gold Kit (Epicenter) and rRNA-depleted RNAs were then used to prepare the RNA-seq libraries with the ScriptSeq v2 kit. Library quality was assessed using a DNA high sensitivity chip on a Bioanalyzer 2100.

### Transcriptome sequencing and Bioinformatics analyses

Libraries were sequenced on an Illumina platform with 75bp paired-end reads. Read quality was assessed with FastQC v0.11.7 (https://www.bioinformatics.babraham.ac.uk/projects/fastqc/). Reads were first aligned against the *wBm* genome (NC_006833) and *B*. *malayi* mitochondrial (NC_004298) with Bowtie2 v2.3.5.1[55] using the following options:—*very-sensitive-local—no-mixed—no-discordant*. Unmapped reads were then aligned against the *B*. *malayi* genome version 4.0 (parasite.wormbase.org) with the splice aware aligner HISAT2 v2.1.0[56] using the following parameters:—*no-mixed—no-discordant*. After alignment files manipulation with Samtools version 1.8[57], read counts were assigned to each genomic feature (CDSs or rRNA) with featureCounts from subread v1.6.2[58] with standard parameters using *w*Bm annotation accessed at NCBI in September 2018, *B*. *malayi* mitochondrial annotation accessed at NCBI in September 2019 and *B*. *malayi* annotation WS265 (parasite.wormbase.org). Significant differentially expressed genes were identified with DESeq2 v1.22.2[59] using a threshold of FDR<0.01 and a log_2_ fold change +/- 2. GO-term enrichment analysis was performed on the interactive web application iDEP (integrated Differential Expression and Pathway analysis) v9.0[60].

### Validation of Gene Expression level by RT-PCR and qRT-PCR

The differential expression of *B*. *malayi* and *Wolbachia wBm* genes in the different tissues was confirmed by RT-PCR for PZ and by qRT-PCR for MZ and BW (S1 Fig). Tissue collection and RNA extraction were performed as described above. First strand cDNA synthesis was performed with the SuperScript VILO cDNA Synthesis Kit (ThermoFisher Scientific) from 100ng total RNA following the standard protocol. RT-PCR was performed on a Bio Rad T10 Thermal Cycler with NEB OneTaq DNA Polymerase following manufacturer protocols with a Tm of 55°C and for 25 cycles for three biological replicates. *cdk-1* (WBGene00230411; 531bp-long amplified fragment) was used as a PZ-specific gene; *rps-4* (WBGene00226794; 583bp) as a housekeeping gene. *wBm 16S* (WBM_RS02885; 424bp) was used as a positive control of *Wolbachia* infection (see primer sequence list in S5 Table).

Quantitative RT-PCR (qRT-PCR) was performed on a Stratagene Mx3000P for three biological replicates and three technical replicates for each analyzed tissue. Gene expression was estimated using the standard ‘ΔΔCt’ method. For internal control of *B*. *malayi* gene expression, we selected two genes coding for *rpl-12* (WBGene00227210) and *rps-4* (WBGene00226794) because of their constitutive expression in all samples according to the RNA-seq data. WBGene00222423 and WBGene00222275 were used as tissue- specific targets for MZ and BW, respectively (see primer sequence in S5 Table).

### Comparative genomics

In order to better define the genetic features of the *B*. *malayi* genome, ranging from those genes shared by all species within the Opisthokonta, to the evolutionary innovations unique to the *Brugia* species, we created an orthology map of the *B*. *malayi* genome using data hosted in the Wormbase Parasite Compara database. To obtain a thorough view of the *B*. *malayi* orthology we selected 24 nematode species genomes (*Brugia pahangi*, *Brugia timori*, *Wuchereria bancrofti*, *Loa loa*, *Litomosoides sigmodontis*, *Onchocerca flexuosa*, *Onchocerca ochengi*, *Onchocerca volvulus*, *Dirofilaria immitis*, *Ascaris suum*, *Strongyloides ratti*, *Panagrellus redivivus*, *Bursaphelenchus xylophilus*, *Globodera pallida*, *Meloidogyne hapla*, *Meloidogyne incognita*, *Pristionchus pacificus*, *Haemonchus contortus*, *Heterorhabditis bacteriophora*, *Necator americanus*, *Caenorhabditis elegans*, *Romanomermis culicivorax*, *Trichinella spiralis and Trichuris muris*) spanning the entire nematode phylogeny [61], the tapeworm *Taenia saginata* and five outgroup model organisms (*Drosophila melanogaster*, *Homo sapiens*, *Mus musculus*, *Danio rerio* and *Saccharomyces cerevisiae*). We then collated protein homology values for each of the 10,922 *B*. *malayi* genes present in the Compara database in each of the 30 species described above. The detailed methodology and pipeline implemented to produce the Wormbase Parasite Compara database has been described previously (https://parasite.wormbase.org/info/Browsing/compara/index.html) and (http://www.ensembl.org/info/genome/compara/homology_method.html). We interrogated the Wormbase Parasite Compara database in June 2019 via the biomaRt library implemented in R [62]. We then used the WormBase ParaSite REST API Endpoints to obtain the root clade of each gene tree phylogeny. Circular maps harboring all the *B*. *malayi* gene orthologies along with their clade root origin were made with Circos v0.69–6 [63].

To classify genes using both their expression patterns and their phylogenetic origin, we selected genes specifically upregulated in each of the three tissues. We then conducted enrichment analyses (Fisher’s exact test) of those tissue-specific or developmental stage-specific upregulated genes for each of the clade root categories of the whole *B*. *malayi* orthology classification.

### Immunostaining and microscopy

Frozen *B*. *malayi* female specimens were thawed and fixed in paraformaldehyde (3.2% in PBS) for 10 min, then kept in PBS until dissection. Dissected ovaries, as well as hypodermal fragments were then freeze-cracked as described [30], and kept in cold methanol for 10 minutes at -20C. Upon complete methanol evaporation at room temperature, the anti *wBm* antibody (NR-31029, monoclonal Anti-*Wolbachia* Surface Protein, Bei Resources) was added to the samples in PBS at 1/250 overnight at room temperature. After three washes of at least 5 min each, a secondary fluorescent antibody (Alexa fluor 488 anti-mouse, Invitrogen) was added at 1/500 and left overnight at room temperature, then rinsed three times with PBS, and slides were mounted with DAPI Vectashield. Images were acquired using a Leica TCS SP5 confocal microscope with a 63X objective.

## Supporting information

S1 Fig Validation of differential gene expressions in PZ, MZ and BW RNAseq analyses cDNAs were produced using total RNA extracted from pools of 60 PZ, 60 MZ and 30 BW fragments for each of the three biological replicates. (A) Semi-quantitative PCR validation of PZ specific gene expression. The gene specific to the PZ region is *cdk1* (WBGene00230411; 531bp) and shows expression only in the PZ triplicates. Housekeeping gene is *rps-4* (WBGene00226794; 583bp) and a positive control of *Wolbachia* infection is 16S (WBM_RS02885; 424bp). (B) qRT-PCR validation of MZ or BW specific gene expressions. The following genes were selected: WBGene00222275 (upregulated in RNAseq in BW, green), WBGene00222423 (up-regulated in RNAseq in MZ, orange), with WBGene00226794 (*rps*-*4*) as an internal control. The Y axis corresponds to the log_2_ fold change of gene expression in BW compared to MZ; X-axis to the BW and MZ biological triplicates.
(TIF)

S1 Table *wBm* gene expression data in PZ, MZ and BW, compared between samples as follows: PZ vs MZ; PZ vs BW; MZ vs BW. Expression values are color-coded from low expression level (green) to high expression level (red). Fold change values are color-coded in green for a log_2_ fold change < -2 and in red for a log_2_ fold change > 2. FDR are color-coded in pink when < 0.01.
(XLSX)

S2 Table *B*. *malayi* gene expression data in PZ, MZ and BW, compared between samples as follows: PZ vs MZ; PZ vs BW; MZ vs BW. Expression values are color-coded from low expression level (green) to high expression level (red). Fold change values are color coded in green for a log_2_ fold change < -2 and in red for a log_2_ fold change > 2. FDR are color coded in pink when < 0.01.
(XLSX)

S3 Table *B*. *malayi* expression data of genes specifically up regulated in PZ and MZ. Expression values are color-coded from low expression level (green) to high expression level (red). Fold change values are color coded in green for a log_2_ fold change < -2 and in red for a log_2_ fold change > 2. FDR are color coded in pink when p-value < 0.01.
(XLSX)

S4 Table List of genes without Wormbase ID or not present in the Compara database. (XLSX)

S5 Table List of primers used in RT-PCR and qRT-PCR to confirm selected gene expression levels of RNAseq data. (XLSX)
