# Supplementary figures and images for: Dual RNAseq analyses at soma and germline levels reveal evolutionary innovations in the elephantiasis-agent Brugia malayi, and adaptation of its Wolbachia endosymbionts
Source: PLoS Negl Trop Dis. 2021 Jan 6;15(1):e0008935. doi: 10.1371/journal.pntd.0008935 (PMC7787461; doi:10.1371/journal.pntd.0008935)

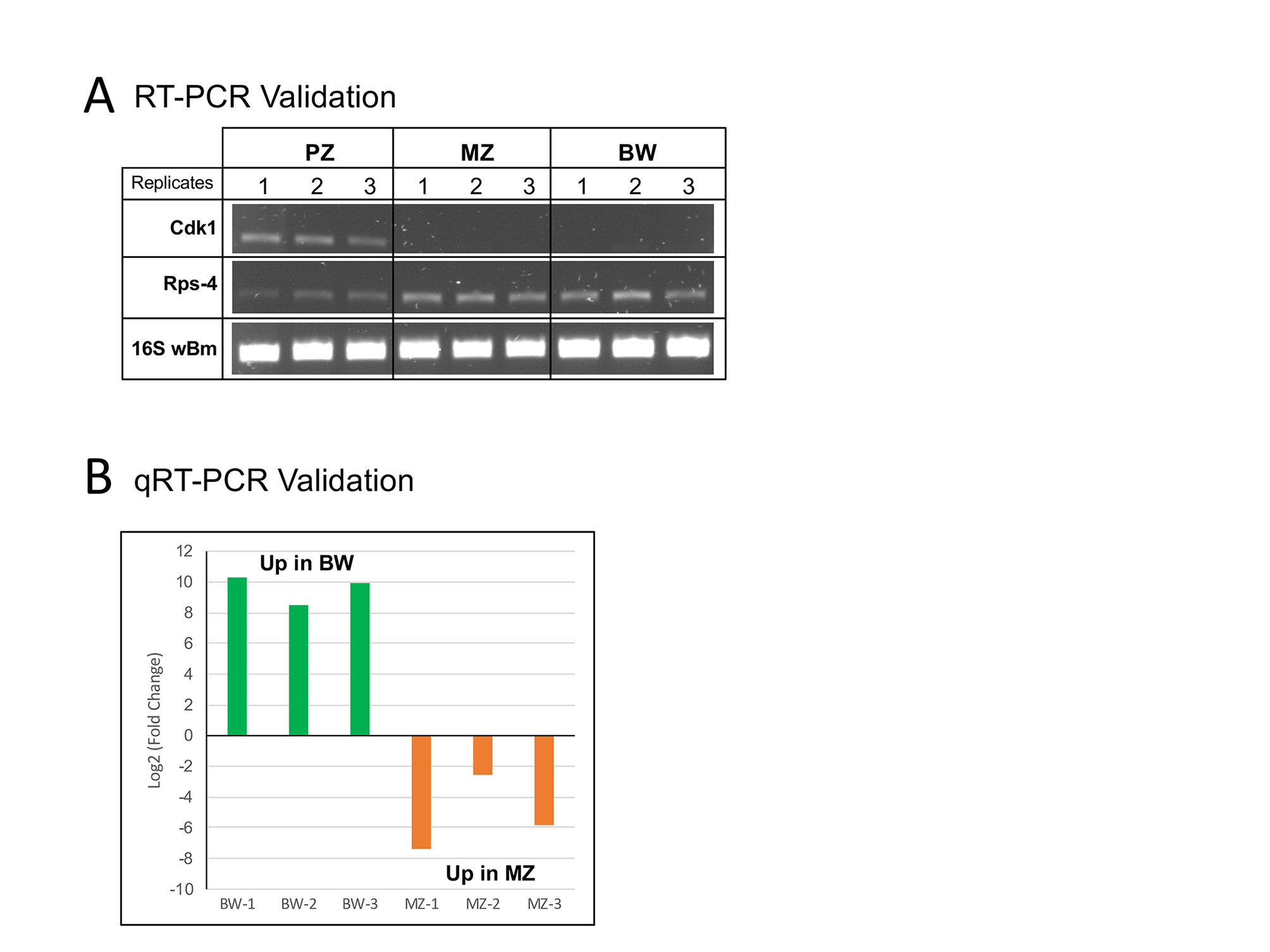

Supplement: S1 Fig — (A) Semi-quantitative PCR validation of PZ specific gene expression. The gene specific to the PZ region is cdk1 (WBGene00230411; 531bp) and shows expression only in the PZ triplicates. Housekeeping gene is rps-4 (WBGene00226794; 583bp) and a positive control of Wolbachia infection is 16S (WBM_RS02885; 424bp). (B) qRT-PCR validation of MZ or BW specific gene expressions. The following genes were selected: WBGene00222275 (upregulated in RNAseq in BW, green), WBGene00222423 (up-regulated in RNAseq in MZ, orange), with WBGene00226794 (rps-4) as an internal control. The Y axis corresponds to the log2 fold change of gene expression in BW compared to MZ; X-axis to the BW and MZ biological triplicates. (TIF) [file pntd.0008935.s001.tif]
